# Mechanism of Viral Suppression among HIV Elite Controllers and Long-Term Nonprogressors in Nigeria and South Africa

**DOI:** 10.3390/v14061270

**Published:** 2022-06-10

**Authors:** Rahaman Ademolu Ahmed, Khalid Olajide Adekoya, Chika Kingsley Onwuamah, Bolanle Olufunmilayo Oboh, Smita Swaminathan Iyer, Ayomide Samuel Oluwatosin, Rosemary Ajuma Audu, Oliver Chukwujekwu Ezechi

**Affiliations:** 1 Cell Biology and Genetics Department, University of Lagos, Lagos 101017, Nigeria; kadekoya@unilag.edu.ng (K.O.A.); boboh@unilag.edu.ng (B.O.O.); 2 Center for Human Virology and Genomics, Microbiology Department, Nigerian Institute of Medical Research, Lagos 100001, Nigeria; chikaonwuamah@yahoo.com (C.K.O.); ayomidetosin@gmail.com (A.S.O.); rosemaryaudu@yahoo.com (R.A.A.); 3 The Center for Immunology and Infectious Diseases, University of California, Davis, CA 95616, USA; smiyer@ucdavis.edu; 4 Clinical Science Department, Nigerian Institute of Medical Research, Lagos 100001, Nigeria; oezechi@yahoo.co.uk

**Keywords:** HIV elite controllers, viraemic controllers, long-term nonprogressors, Nigeria, South Africa

## Abstract

A subgroup among people living with HIV (PLHIV) experience viral suppression, sometimes to an undetectable level in the blood and/or are able to maintain a healthy CD4+ T-cell count without the influence of antiretroviral (ARV) therapy. One out of three hundred PLHIV fall into this category, and a large sample of this group can be found in areas with a high prevalence of HIV infection such as Nigeria and South Africa. Understanding the mechanism underpinning the nonprogressive phenotype in this subgroup may provide insights into the control of the global HIV epidemic. This work provides mechanisms of the elite control and nonprogressive phenotype among PLHIV in Nigeria and South Africa and identifies research gaps that will contribute to a better understanding on HIV controllers among PLHIV.

## 1. Background

Over 38 million people are living with HIV/AIDS globally, while an estimated 2.6 million are added annually [1,2]. There were 1.5 million new HIV infection cases in 2020 with over sixty percent recorded in sub-Saharan Africa [1]. Nigeria and South Africa harbor an immense health burden of HIV infection in Africa and are among the countries of utmost concern in the context of the world’s HIV challenges [3,4].

**Figure 1:**
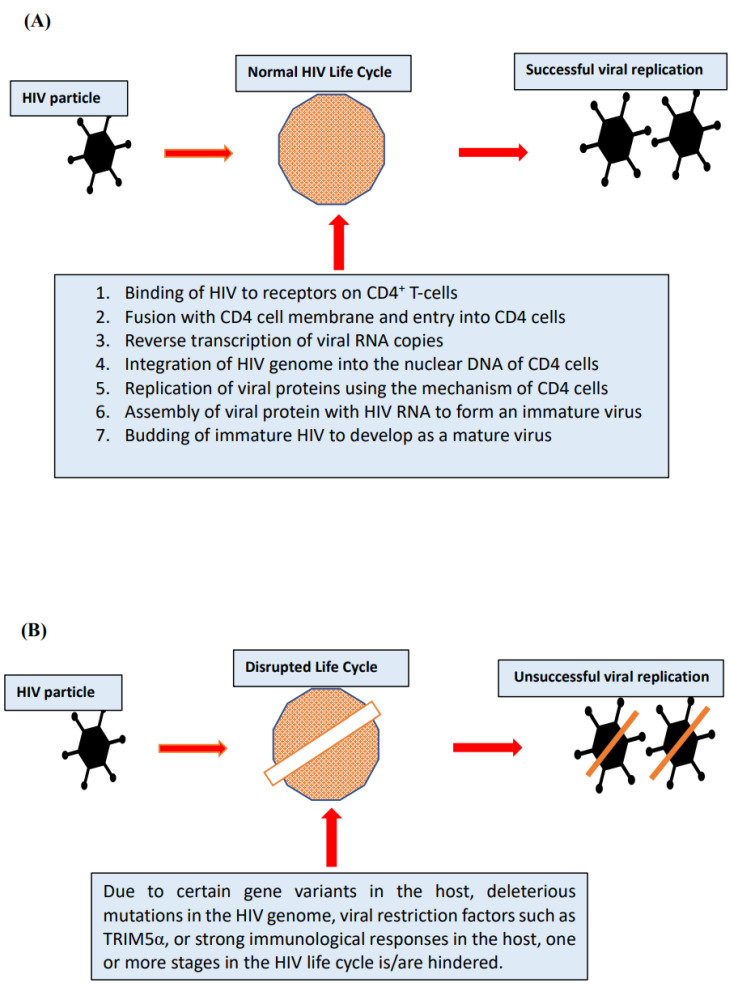
Understanding the mechanism of viral suppression and favorable disease outcomes among HIV elite controllers and other HIV controllers. (**A**) Without the influence of barriers, such as antiretroviral drugs, or unique features that contribute to viral suppression among the elite controllers, viraemic controllers, or long-term nonprogressors, HIV replicates successfully and infects new CD4^+^ T cells. This eventually causes a high viral load in the host system. (**B**) In HIV controllers, such as elite controllers and viraemic controllers, one or more of the stages of HIV’s life cycle is inhibited or interrupted by (i) host unique features such as potent immune responses that are capable of effectively neutralizing, engulfing, or lysing HIV-infected cells; favorable genetic variants that do not support HIV replication; viral restriction factors that are host-acting proteins that provide first-line protection against infection of new host cells; (ii) viral factor, specifically, deleterious mutations in the HIV genome that may cause loss of fitness during viral replication. The mechanism of the nonprogressive phenotype in HIV infection can therefore be explicitly delineated when specific features that favor positive disease outcomes among the HIV controllers are identified.

Before the recent clinical recommendation [5] that requires all detected HIV-infected individuals to initiate antiretroviral therapy (ART), variation occurred among people living with HIV (PLHIV) regarding the progression of HIV infection or replication of viral RNA, on the basis of which some were recommended for antiretroviral therapy (ART), while some who were able to control viral replication and/or disease progression would not initiate ART [6,7,8]. Patients who without ART treatment were able to sustain a stable CD4+ T-cell counts within a healthy range (above 450 cells/µL blood) and remain asymptomatic for a long period of time were generally referred to as long-term nonprogressors (LTNPs) [7,9], but a subgroup among the LTNPs, known as slow progressors (SPs), exhibited a high viral load despite manifesting a long-term nonprogressive phenotype [10]. Viraemic controllers (VCs) were able to contain a viral load at a level below 2000 HIV-RNA copies/mL of plasma and maintain a healthy CD4+ T-cell count for a prolonged length of time without ART therapy [8,11]. Some, however, had the ability to suppress viral replication to an undetectable level (<50 HIV-RNA copies/mL plasma) without ART intervention and were referred to as elite controllers (ECs) [7,12,13]. This HIV-control phenomenon can be influenced by factors of host genetics and immunity as well as viral features that may contribute to HIV suppression [7,13]. The general mechanism of this phenomenon is illustrated in Figure 1. Adequate investigation into this subgroup may channel a route for better therapeutic interventions and provide bases for efficacious HIV vaccine development [8,13,14,15].

One of the factors that contributes to virological suppression among ECs and LTNPs is the transmission of attenuated HIV particles from previous nonprogressors (who may have also inherited such less virulent strain through vertical infection) to new individuals who also develop similar clinical characteristics [8,16,17,18,19]. For example, Casado and colleagues isolated HIV-1 from five elite controllers, who were all Spanish and with similar clinical and epidemiological features, to identify viral correlates of the elite control phenotype. Eleven amino acid mutations were identified across the isolates and were attributed to a common feature that enhanced viral suppression among the five individuals [16]. In a further investigation to identify the mechanism underlying the positive clinical feature in the Spanish cohort, the HIV Env isolated from the ECs were incapable of infecting CD4+ T cells and exhibited a loose actin/tubulin cytoskeleton configuration compared to the properties of Env that were isolated from rapid progressors [17]. However, the characteristic viral suppression experienced by most ECs is attributed to post-infection development rather than the viral factor being the primary correlate of nonprogressive HIV infection [20,21,22,23,24,25]. In this case, the hosts are infected with HIV of spontaneous replicative capacity (fully competent virus), but the unique characteristics that the hosts possess inhibit virological replication, and the hosts eventually develop as HIV controllers [22]. This is evident from transmission pair studies where HIV was transmitted from a rapid progressor to a new individual who later became an EC, or where isolated virus from two different individuals exhibited full viral replicative competency in vitro, but one of the hosts was able to naturally control replication in vivo using the host-related mechanisms, while the other developed as a rapid progressor [21,26,27]. Some HIV controllers also progress later in life to develop as rapid progressors, thereby losing virologic control of HIV infection. This emphasizes the fact that such ECs or VCs were infected with a replication-competent virus that was initially suppressed using the host genome and the immune response mechanism, but when they lose the driving factor behind their virologic control, they also lose the associated favorable disease outcomes that are attributed to HIV controllers [21,24,27,28].

Gag-specific CD4+ and CD8+ T cells have been attributed to have effective immune responses and low viral escape characteristics, enhancing strong immune capture of HIV-infected cells thereby inhibiting viral replication [8,29,30,31]. The high frequency of Gag-restricted CD4+ and CD8+ T cells has been well linked to the elite control phenotype in HIV infection, and this could be associated with qualitatively robust immune responses that enhance efficient viral suppression [8,29,32]. Furthermore, helper T cells exhibited by HIV controllers possess superior qualitative properties that enhance effective viral suppression better than the characteristic helper functions demonstrated by the rapid progressors [32]. Some ECs and LTNPs are also able to develop broadly neutralizing antibodies for diverse HIV strains, thereby exhibiting strong adaptive immune responses to clear HIV-infected cells and, eventually, suppressing viral replication [33,34].

The mechanism of the host’s innate immunity toward achieving a nonprogressive phenotype, as exhibited by HIV controllers, involves, among others, the recruitment of an elevated number of correlating dendritic cells, macrophages, natural killer T cells, monocytes, or natural killer cells [24,32,35]. The high frequency of these innate immune cells is achieved and maintained by an interdependence signaling among the cells and through the action of cytokines, such as IL-12, IL-15, IFN-α, and IFN-β, which partially or together play inhibitory roles in viral replication [31,32,35,36,37,38,39]. Furthermore, the abundant expression of certain viral restriction factors, which in connection with the immune system disrupts one or more stages of the HIV life cycle, has also been identified as one of the underlying mechanisms of viral suppression among ECs and LTNPs [8,40]. Viral restriction factors that have been reported in relation to nonprogressive HIV infection include, among others, the sterile alpha motif and histidine-aspartate domain 1 (SAMHD1), tetherin, APOBEC3G, TRIM-5α, serin incorporator 3/5 (SERINC3 and SERINC5), B-lymphocyte-induced maturation protein-1 (BLIMP-1), endoplasmic reticulum class I α-mannosidase (ERManI), translocator protein (TSPO), guanylate-binding protein 5 (GBP5), and zinc-finger antiviral protein (ZAP) [8,40,41,42]. Mutations in the genetic loci of these antiviral factors may also play important roles in the upregulation or underexpression of the respective restriction factors. However, they are expected to contribute to viral suppression when they are upregulated [8,41]. In this case, the mechanism of virologic control is associated with the host genome characteristics.

The host genome feature that has been widely and most significantly reported as a correlate of the nonprogressive phenotype is the abundant representation of certain human leukocyte antigen (HLA) alleles, which are mostly found especially among ECs. The most correlating HLA alleles for elite control of HIV infection are HLA-B*57 and -B*27 [32,43,44,45,46,47], although -B*58:01 has also been reported as a correlate of the nonprogressive phenotype in HIV infection as reported in two recent reviews [48,49]. The cytoplasmic domains of HLA-B alleles show higher resistance to Nef-enhanced immune disruption (on the antigen-presenting cells) compared to the HLA-A alleles; therefore, HLA-B alleles enhance better cytotoxic T-lymphocyte (CTL) recognition of HIV-infected cells, thereby enhancing optimum destruction of infected cells and eventually suppressing viral replication in HIV nonprogressors [29,46,49]. Although viral escape from the unique HLA-B-specific CTLs among ECs has been reported, such escaped viruses often lose replicative vigor and, therefore, may not contribute to effective viral multiplication [46,50,51].

The presence of a heterozygous 32-base pair deletion in the CCR5 gene (CCR5-Δ32) is another feature of host genome characteristics among ECs and other LTNPs [47]. Considering the preventive effect of the homozygous CCR5-Δ32 genotype in HIV infection, inhibiting the successful binding of HIV to CD4+ T cells [52], the presence of heterozygous CCR5-Δ32 in an HIV patient is therefore expected to reduce the rate of viral replication, thereby enhancing positive disease outcomes similar to the clinical experience of ECs and LTNPs [8]. Studies have reported the presence of the CCR5-Δ32 allele in certain HIV nonprogressors [47,53]; however, several others did not find that this feature correlated with nonprogressive HIV infection [54,55]. Other gene polymorphisms, including those of viral restriction factors, such as the Toll-like receptor-9 (TLR-9), stromal cell-derived factor 1 (SDF-1), BST2, killer cell immunoglobulin-like receptors (KIR2), beta-defensin-I, and the APOBEC3G gene, have also been associated with natural HIV control among ECs and LTNPs [8,41,56,57,58].

Due to the large expected population of individuals with the nonprogressive phenotype in regions of high HIV prevalence, such as Nigeria and South Africa, correlates of the nonprogressive phenotype among HIV controllers can be further investigated in such localities to better delineate the mechanism underpinning the positive clinical characteristics [59]. Unfortunately, with the recent clinical recommendation [5], all HIV seropositive individuals are to initiate ART therapies irrespective of HIV viral load or CD4+ T-cell count. Therefore, there is a need to evaluate what has been reported on this unique group and quickly fill research gaps where possible, before the samples of nonprogressors (which may still be presently persevered) are no longer available. This study reviewed reports on elite controllers, viraemic controllers, and long-term nonprogressors in Nigeria and South Africa and identified research gaps that are yet to be filled in these two countries on these subjects.

## 2. Method

An electronic literature search was conducted by querying articles written in English from the PubMed database and the African Journal Online, published between January 1985 and June 2021. Search terms included HIV controller/ or elite controller/ or viraemic controller/ or long-term non progressor/ or slow progressor/ and HIV infection/ or prevalence/ or mechanism of control/ or immune response/ and Nigeria/ or /South Africa. Extracted information from the retrieved articles were grouped into the following headings: (a) low transmission risk of HIV infection from ECs; (b) mechanism delineated from viral features that were associated with nonprogressive HIV infection among ECs and LTNPs; (c) putative mechanism of viral control as influenced by the host genome; (d) mechanism of viral control among ECs and LTNPs as derived from associated host immune characteristics.

## 3. Low Transmission Risk of HIV Infection from ECs

To determine the transmission risk of HIV infection from elite controllers via blood medium, Vermeulen et al. [60] evaluated viral load count in blood samples donated by elite controllers and by HIV-infected individuals who were at the window stage (WP; when a patient has not developed an antibody response to the infection) at the time of donation. Samples were obtained from the South African Blood Service (SANBS) after 3 years in the repository. The authors identified a 50% minimum infectious dose (ID50 which is the number of virus particles that is required to initiate infection in 50% of normal adult humans exposed by a given route) from the evaluated viral load and utilized Poisson statistics to determine the transmission risk of HIV infection in the two categories. Their results revealed that a maximum viral load in EC blood donation was 5.5 copies/mL while that from individuals at WP was up to 500,000 copies/mL. It was estimated that only 2.2% of the blood from the EC samples could successfully transmit HIV infection during transfusion with an ID50 of 350 virions, while up to 15% could effectively transmit from samples donated by individuals who were at the window period. However, Sykes et al. [61] evaluated false EC samples obtained from blood donors at the South African National Blood Service (SANBS): those who did not disclose ART use during the predonation interview and had an undetected HIV viral load were all presumptively categorized as elite controllers. Plasma samples for all presumed ECs between 2010 and 2016 were tested for ART drugs using validated assays on liquid chromatography-tandem mass spectrometry. Approximately two-thirds (66.4%, *n* = 150) of the presumptive EC samples tested positive for ART drugs, indicating a high proportion of false EC status within the seven-year period. Therefore, this suggests that elite control samples should be evaluated for ART presence before utilizing them for studies that aim to identify correlates of the nonprogressive phenotype in HIV infection.

Furthermore, Ferrand et al. [62] gathered existing population data including crude birth and death rates from the Population Division of the United Nations (UNIPOP) database with respect to before (i.e., 1980) and factored in antenatal HIV prevalence from southern African countries (i.e., South Africa, Swaziland, and Zimbabwe). These data were used to estimate the prevalence and mortality of fast and slow progressors among children infected with HIV. The result of their model revealed that HIV prevalence among children was expected to decrease in Zimbabwe but increase in South Africa. Children who were infected after birth through breastfeeding were likely to be slow progressors compared to children infected in the uterus before birth. However, death among untreated slow progressors was expected to increase in South Africa by over 300% by 2030. Fortunately, since 2016, all diagnosed cases of HIV infection, irrespective of the level of disease progression, must initiate ART [5], thereby reducing the expected rise in mortality among SPs.

## 4. Mechanism Delineated from Viral Features That Were Associated with Nonprogressive HIV Infection among ECs and LTNPs

The genome of the HIV-1 virus comprises nine genes that code for fifteen viral proteins. The encoded regions when expressed develop into viral structural proteins, essential regulatory elements, or accessory regulatory proteins [63,64]. HIV-1 structural proteins consist of group-specific antigens (Gag), polyproteins (which make up the viral core), viral enzymes (Pol), and the envelope (Env). The envelope gene locus encodes gp160, a glycoprotein, which when cleaved forms two mature protein subunits (i.e., gp41 and gp120) found on the surface of the virus and are important for the successful binding and initiation of infection in the host cells [64,65,66,67,68]. Both gp120 and gp40 have glycosylated nucleotide sites that are used to mask the viral envelope from host immune detection [65,68,69].

Considering the importance of this HIV envelope in the initiation and progression of HIV infection, gp160 has been one of the targets of HIV vaccine development [70,71,72]. Archary et al. [73] investigated the differences in HIV gp160 dynamics between HIV LTNPs and rapid progressors. Retrospective samples of eight antiretroviral-naive HIV-1 clade C patients from the Sinikithemba cohort in Durban, South Africa, were used for this investigation with a follow-up history of up to 39.8 months. The CD4+ T-cell count and viral load were used as parameters to categorize participants as LTNPs (>500 CD4+ cell counts and less than 10,000 viral RNA copies/mL) or progressors (below 500 CD4+ cell counts and above 10,000 viral RNA copies/mL), while single-genome amplification and sequencing were used to study the dynamics of the gp160 protein including diversity, divergence, length of the constant (C) and variable (V) regions, as well as the putative N-linked glycosylation sites (PNGs). Their findings revealed that nucleotide diversity in the constant regions, C2 and C3 as well as in the hypervariable region V3, were significantly higher in LTNPs than in rapid progressors. Furthermore, increased amino acid length in the variable regions (V1-V4) and fewer N-linked glycosylation sites on the gp120 locus were observed among LTNPs, while the gp41 locus was significantly longer with fewer PNGs when compared with rapid progressors. The regions of V1, V4, C3, C4, and gp41 mapped positively in LTNPs, while only gp41 did for progressors. The high diversity in the C2, C3, and, especially, in the V3 regions among LTNPs was suggested to be associated with ART-free suppression of viral replication. The different dynamics of the HIV-1 envelope found among the LTNPs and the rapid progressors revealed unique patterns for each of the two subgroups, which according to the authors will support the understanding of the viral control mechanism, harnessable for effective HIV vaccine development. Other studies that investigated viral features as corelates of nonprogressive infection, but largely as an effect of robust immune responses among LTNPs, are discussed in relation to their causal response under the host immunity section.

## 5. Putative Mechanism of Viral Control as Influenced by the Host Genome

Chemokine receptors are essential for the progression of several human diseases and are crucial for effective viral transmission and replication in HIV infection [74,75,76,77,78]. The chemokine coreceptors that are commonly utilized for HIV infection are C-C chemokine receptor type 5 (CCR5) and C-X-C chemokine receptor type 4 (CXCR4) [78,79,80], although CXCR6 has also been reported as a coreceptor for HIV infection [81,82]. Genetic variation in the chemokine receptor loci may have an impact on host susceptibility to HIV infection or on the rate of progression of viral replication [80,81,83].

Pincton et al. [84] studied the genetic variation of a chemokine receptor (CXCR6) among HIV-1-infected, ART-naive Black individuals from Soweto and Johannesburg using rs2234358 and rs2234355 SNPs. These SNPs have previously been associated with disease progression and viral suppression among HIV-1-infected patients [85,86]. Eleven HIV ECs, thirty VCs (with a viral load below 2000 RNA copies/mL and a CD4 count above 500 cells/μL), eleven high viral load LTNPs (without significant CD4+ T-cell decline for up to seven years despite having a viral load greater than 10,000 RNA copies/mL and a CD4 count less than 500), and seventy-two rapid progressors were recruited for the study. The CXCR6 region was sequenced from genomic DNA and analyzed for the presence of single-nucleotide polymorphisms (SNPs) and indels using SEQUECHER software (version 4.5). The authors found that allele rs2234358-T was significantly underrepresented among VCs compared to high viral load LTNPs and fast progressors; the homozygous rs2234358-T allele (TT) was also underrepresented among VCs than the progressors and healthy controllers. The genotype rs223455-GA was also found to be overrepresented in VCs compared to healthy controllers and progressors. The authors, therefore, identified the two CXCR6 SNPs (i.e., rs2234358 and rs2234355) as factors contributing to the viraemic control of HIV infection in South Africa. Choge et al. [87] also investigated the use of CXCR4 and CCR5 chemokine coreceptors by HIV-1 subtype C virus isolated from fast progressors and LTNPs using heteroduplex mobility assays. Their study revealed that 88% of isolates from LTNPs used CCR5 only (similar to fast progressors with 93%), 8% used CXCR4 only, and 4% used both CCR5 and CXCR4. However, two children with the virus that was identified for CXCR4 usage progressed to AIDS. The authors concluded that an HIV virus that uses CCR5 is common among both progressors and slow LTNPs, while that of CXCR4 is generally uncommon, and there was no clear distinction between LTNPs and rapid progressors based on chemokine receptor type that was used for infection of new host cells.

Compared to the frequency among people living with HIV who fall into the category of rapid progressors, higher or overexpression of certain viral restriction factors that were previously mentioned have been associated with positive disease outcome among ECs and LTNPs [8,40,42]. However, if the viral inhibitory mechanism is enhanced by certain polymorphisms in the regulatory or other regions of the respective gene, the mechanism of viral control will be attributed to the host genome features. Therefore, for this review, the harmonized viral restriction factors that fell under the category of the host genome characteristics are also reported in this section. Two single-nucleotide polymorphisms (i.e., rs2072254 and rs2072255) in the RICH2 gene have been linked to viral progression in HIV infection among Caucasians [88]. Paximadis et al. [89] studied the association of these SNPs with HIV control in elite controllers and LTNPs among Black South Africans. Viral RNA was quantified, and CD4+ T cells were counted and documented for each HIV-infected patient, while the RICH2 gene was amplified and sequenced to analyze for single-nucleotide polymorphisms. The authors found no significant difference in the allelic and genotypic frequencies between any category of LTNPs and rapid progressors. However, there was a combination trend of rs2072254AA and rs2072255GA being significantly underrepresented among ECs and VCs compared to the healthy controls. In addition, a low CD4+ T-cell count was associated with the combined genotypes: rs2072254AA/rs2072255GA and rs2072255 (GA+AA). Following these significant allelic and genotypic combinations in HIV controllers and the same pattern of low disequilibrium linkage of these SNPs among Black Africans in the 1000 genome project, the authors concluded that the two SNPs may be associated with the natural control of HIV-1 in Black sub-Saharan Africans.

The bone-marrow stromal cell antigen 2 (BST2), also known as tetherin, is a viral restriction factor capable of preventing the release and progression of the enveloped virus in mammalian hosts [90,91]. Some variants of the BST2 gene have also been correlated with HIV infection and the course of disease progression [92,93]. A study investigated the association of four BST2 SNPs (i.e., rs3217318, rs12609479, rs10415893, and rs113189798, previously correlated with HIV-1) with viral suppression among ART naive Black South Africans [94]. Among the BST2 variants, heterozygous rs113189798-A/G was prevalent among ECs compared to the rapid progressors, while rs113189798-GG showed a significant correlation with viral suppression. The combined genotype of rs3217318(i19/Δ19), rs12609479(G/G), rs10415893(G/A), and rs113189798(A/G) were also associated with high CD4+ T-cell count among rapid progressors. However, a high rate of CD4+ T-cell decline was associated with the heterozygous rs3217318 indel (Δ19/i19), corroborating a report from a Spanish HIV cohort [92]. The putative mechanism behind this feature was that since the associated variant is in a promoter region, the rs3217318 indel (Δ19/i19) may suppress BST2 expression, thereby reducing the viral inhibiting factor available for attachment to the HIV domain and, eventually, enhancing rapid viral replication among fast progressors. The reverse mechanism would promote the nonprogressive phenotype as observed in ECs or VCs.

Furthermore, the most significant host genome corelates of natural HIV control among ECs and several other LTNPs is the HLA polymorphism and its respective abundance that is required for a robust and effective immune response necessary for nonprogressive HIV infection. However, because of their important and associated role in the mechanism underpinning host immunity for viral suppression, this feature is described largely under the host immunity section.

## 6. Mechanism of Viral Control among ECs and LTNPs as Derived from Associated Host Immune Characteristics

The primary target of HIV infection is CD4+ T cells, resulting in the massive loss of CD4+ T cells during the early stage of infection, especially in the lymphoid tissue that is associated with the gut [67,95,96]. CD8+ T cells are, however, activated as a host immunological response to elicit cytotoxic effects against HIV infection at this acute stage [97,98,99]. Gray et al. [100] investigated the patterns of HIV-1-specific T cells at the acute stage of infection using the gamma interferon (IFN-gamma) enzyme-linked immunospot (ELISPOT) assay to measure the level of responses against the HIV-1 proteome. This was aimed at understanding the early patterns of responses to specific HIV components during the progression of HIV infection. These data were further utilized to investigate the specific paths of responses in VCs and LTNPs recruited in a CAPRISA (Centre for the AIDS Program of Research in South Africa) study. The authors found the viral proteins, Nef (negative factor, which is essential for HIV replication), Gag (a group-specific antigen that develops to the proteins of the viral core), and Pol (which develops to form the viral enzymes needed for viral effective activities in the host), more dominant than other viral proteins. The T-cell responses for Nef were higher than the other viral proteins but not related to the course of viremia, although there was a nonsignificant trend of an increased breadth of response with viral load. Rapid progressors, however, possessed a diverse epitope recognition pattern compared to the LTNPs. This finding is similar to a report by MLotshwa et al. [101], who also investigated the associated pattern using 53 infected individuals also from a CAPRISA study. They found Pol, Nef, and Gag, predominantly recognized during the course of progression, and Nef responses were the fastest to evolve compared to the other viral proteins. However, in that study, Gag was associated with viral control, and there was a 23% chance of an increased response weekly to the Nef protein. Following a categorization of the T-cells in three responses (i.e., persistent, lost, or new responses), rapid progressors showed a significantly low persistent response compared to the LTNPs. The authors, therefore, concluded that persistent T-cell responses necessary to incessantly fight HIV infection at the acute stage are higher among LTNPs, which suggest a reason for the viral suppression during the progression through the chronic stage of infection.

Towards identification and understanding specific T-cell responses responsible for HIV viral suppression among slow progressors and elite controllers, Thobakgale et al. [102] studied the characteristic responses of CD8+ T cells in 15 infants who were perinatally HIV infected starting from the day of birth up to a period of 55 months. The study involved seven slow progressors and eight rapid progressors recruited from two hospitals in Durban, South Africa. Plasma viral load was quantified while T-cell interferon-gamma production was measured using the IFN-gamma ELISPOT assay. T-cell polyfunctionality responses were further evaluated, which defines the ability of HIV-specific CD8+ T cells to produce multiple cytokines and chemokines in response to viral antigens. Their findings revealed no significant difference between the two groups for specific T-cell responses. However, slow progressors significantly expressed four CD8+ T-cell polyfunctional responses (i.e., HIV-1-specific interferon (INF-gamma); cluster of differentiation 107a (CD107a); tumor necrosis factor-alpha (TNF-alpha); macrophage inflammatory protein (MIP-1 beta)) when assessed together. These polyfunctional responses were, therefore, suggested to be associated with viral suppression or slow disease progression in HIV-infected children.

Furthermore, Laher et al. [103] studied the characteristics of CD4+ T-cell responses at the acute stage of infection in 80 HIV-1 (clade C)-infected persons from the Zulu/Xhosa population who were ART-naive at the time of the investigation. The authors first screened the entire proteome of HIV-1 among infected patients from the Zulu ethnic group to identify the most dominant peptides of the HIV-specific CD4+ T-cell epitopes, and they found the Gag epitope was the most prevalent peptide. They further identified the dominant major histocompatibility complex class II DRB1 alleles that were associated with the preidentified immunodominant CD4+ T-cell epitope. The alleles were recognized as DRB1*13:01 and DRB1*11:01. This information was used to synthesize MHC class II tetramers (specific for the Gag peptides), which were used to investigate the frequency and function of the specific CD4+ T-cell responses against HIV infection and progression. Subjects recruited were grouped as HIV controllers (having a viral load < 2000 HIV viral RNA per mL for greater than one year) and chronic progressors (with a viral load > 2000 RNA per mL). The findings revealed that HIV controllers maintain significantly higher frequencies of the MHC class II tetramer associated with specific HIV-specific CD4+ T cells compared to the acute progressors, and they expressed higher cytolytic proteins granzymes A and B by Gag-specific CD4+ T cells. This, according to the authors, suggests that the high frequency of Gag-specific CD4+ T cells may enhance viral suppression by providing a helper function to CD8+ cells, which primarily produce cytolytic signals or directly kill HIV-infected cells.

Moosa et al. [59] investigated the mechanism of viral suppression in two HIV-1 controllers, recruited from the CAPRISA cohort, whose HIV viral copies were undetectable after 10 months and 6 weeks, respectively. They found that both individuals expressed multiple human leukocyte antigen (HLA) class I and II haplotypes (including HLA-B*44:03, -B*81:01, and -DRB1*13 for one of the ECs; HLA-A*74:01, -B*57:03, and -DRB1*13 for the other EC), which have previously been reported to be associated with reduced disease progression [103]. Both subjects also expressed higher p21 mRNA, which has previously been reported to be associated with natural viral suppression and progression to AIDS [104]. The p21 gene codes for a cyclin-dependent inhibitor 1, which triggers the resistance of hematopoietic cells to HIV infection by not allowing for the effective integration of the HIV provirus [105]. The findings of Moosa et al. also revealed that one of the two ECs expressed a high frequency of HIV-specific CD8+ T-cell responses that may trigger a mass cytotoxicity activity on HIV-1-infected cells. The second person, however, had a pre-infection HIV-specific immunity with CD4+ T-cell responses for Gag and Pol, which was identified at a time point of pre-infection, suggested to have strengthened his adaptive immunity against a rapid progression in HIV infection [59].

Furthermore, protective HLA alleles, such as HLA-B*57 and -B*27, which have been reported as significant corelates of the elite control of HIV infection, enhance destruction of HIV infected cells through the cytotoxic effect of CD8+ T-cells [32,44,46]. However, viral suppression experienced by certain HIV controllers may also be influenced by the effect of protective HLA on immune-escaped mutant virus, rendering HIV attenuated for replication in the host [32,46,106]. HLA-B*57, -B*58:01, and -B*81:01 have been reported to induce mutations within Gag epitopes, which can result in reduced viral replication [46,50,107]. A study examined the importance of these protective HLA alleles in viral suppression and reduced disease progression among 61 HIV-infected mother–child pairs in Durban, South Africa [108]. The authors found that slow disease progression was associated with the child or the mother having one of the protective HLA-B alleles, especially when the protective alleles were not shared by both the mother and child. Importantly, the study revealed that mothers with the protective alleles were able to transmit HIV with reduced replicative capacity or low fitness to their children, resulting in low progression of HIV infection. Affirmative to this finding, Prado et al. [109] studied viral characteristics associated with progression among HIV infants from Durban in South Africa. The study’s findings revealed that the slow progression phenotype was related to the HIV strain having mutations that did not support active replication, transmitted from mothers to their children. The mutated variants escaped from the protective HLA Gag-specific epitopes and, eventually, caused the virus to lose replicative vigor [109]. This claim was further confirmed by Tzitzivacos et al. [110], who conducted a full-length genome sequencing on isolated HIV-1 virus from six ART-naive, slow progressor children in Johannesburg. Reports from sequencing revealed that each isolate had at least one mutated or unusual gene or protein that may hinder replicative capacity, resulting in low progression in the children. This low replicative capacity was therefore suggested to be due to the presence of protective HLA-B alleles as initially reported by Thobakgale et al. [108].

## 7. Discussion and Research Gaps on ECS and LTNPS in Nigeria and South Africa

Studies have been conducted and reported from several parts of the world on elite controllers, viraemic controllers, and slow progressors or on heavy viral load long-term nonprogressors that are informing researchers on the mechanism of HIV control and ways to improve the clinical management of the HIV epidemic. Investigations on this subclass of HIV patients can be effectively carried out when an adequate sample size is available as is the case in Nigeria [111] and South Africa [2,4]. Unfortunately, with the recent WHO recommendation, ART must be initiated for all confirmed HIV-positive individuals following the 2016 WHO recommendation [5]; hence, there is a need to harmonize previous reports on this group and identify research gaps that need to be urgently considered before people with these unique features are difficult to identify.

A substantial number of reports have been documented from South Africa on this group of patients including associated transmission risk, protective HLA alleles in HIV infection and other host genome features, associated viral restriction molecules, studies on viral attenuation and its causative factor, as well as correlating immune responses in ECs and LTNPs. The reports were consistent with findings from other parts of the world such as reports on the presence of potent HIV-specific host response [11,112], protective HLA alleles that enhance effective destruction of infected cells, or the release of a mutated virus subtype with a low replicative capacity [50,51,104]. However, reports on viral load rebound following another form of diseased condition or other exposures that may trigger turbulence in the immune response (such as stem cell transplant) were not available from South Africa. This has been reported by investigators in Europe, revealing that a continual rebound of viral load may occur following disruption of body immunity in elite controllers. Smith et al. [113] studied viral reactivation in an elite controller from the United Kingdom (UK) who was treated for myeloma with melphalan and autologous stem cell transplantation. The authors found that the viral load was 28,000 copies/mL on day +13 following transplant with a calculated viral doubling time of 0.5 days or 12 h. Afterward, there was an immune recovery that responded efficiently and suppressed the viral load to fewer than 50 copies/mL by day +37. Furthermore, Watters et al. [114] followed up on the patient and studied the effect of such an immune disruption for a longer period (2.5 years/above 1000 days); they reported the second rebound of viral load on +470 day, phylogenetically different from the first and with a doubling time of 10.5 days, also taken care by an immune response by day +601. The last rebound occurred on day +832. Although the two rebounds were reported to coincide with relapse of myeloma, the kinetics of reactivation and immune recovery may provide an efficient link to developing a successful therapeutic or prophylactic HIV vaccine [10]. This study may, however, be difficult with the present “test and treat” era, where patients should initiate HAART once they are confirmed to be seropositive for HIV infection. Furthermore, there is a need to investigate other viral restriction factors, such as SERINC 3/5, ZAP, ERManI, TSPO, and GBP5, among ECs and LTNPs in South Africa.

Contrarily, to the best of our knowledge, except for a report by Odaibo et al. [111], who documented the prevalence of potential HIV controllers in a part of South West Nigeria, there have been few or no reports on EC, VC, or SP from Nigeria despite its high prevalence of HIV infection. There is a need for researchers to quickly harness suitable preserved samples and records from this group to ensure the availability of regional information for corroboration of studies that have been conducted in other parts of the world. The focus of investigation should include host correlates of HIV control (this will involve favorable gene polymorphisms that contribute to the nonprogressive or slow progressive phenotype, the dynamics of viral restriction factors, and delineating specific innate and adaptive immune responses against HIV progression) and viral features that may promote attenuation or suppress replication of HIV in the host. The research gaps on this unique subject should also be identified and appropriately investigated in the various geographical localities across the globe, thereby enriching the information available on this unique group and together could create a difference in the global fight against the HIV epidemic.

## 8. Conclusions

Various reports have been documented from different parts of South Africa on this subclass of patients in consonance with reports from other parts of the world. Related reports were, however, scarce for Nigeria, and those are necessary to find points of corroboration or to identify differences with reports from other global regions. In order to expand the scope of information globally available on ECs and other LTNPs that may provide the link to further alleviate or totally eradicate the burden of HIV infection, there is a need for researchers to identify related gaps and endeavor to fill the empty spaces in respective localities across the globe.

## 9. What Is Known about This Topic

Sub-Saharan Africa harbors more than 60% of the global health burden due to the presence of HIV infections, with Nigeria and South Africa contributing significantly to this marked burden.

Both countries are expected to have a high prevalence of people with a nonprogressive phenotype for HIV infection.

Identifying the mechanism for viral suppression among the HIV nonprogressors may unravel the path toward control of the HIV epidemic.

## 10. What This Study Adds

This study aimed at harmonizing research findings on elite controllers, viremic controllers, and heavy viral load long-term nonprogressors in South Africa and Nigeria to elucidate the mechanism of the nonprogressive phenotype in the sub-Saharan population.

We harmonized related reports from South Africa, but a large research gap exists on this subject in Nigeria.

It was envisaged that a wide research gap on this subject may also exist in several other populations that may have the capacity to effectively delineate the mechanism underpinning ART-free viral suppression among ECs and LTNPs. Therefore, studies that will merge existing gaps of information are required to better elucidate the mechanism of the nonprogressive phenotype among ECs and LTNPs across respective populations globally. This study should be conducted as soon as possible, using preserved samples of the HIV nonprogressors before the ability to track them and their available storage sources are lost.
